# Res(Uhu)Rge: A Low Cost and Fully Functional Ventilator Indicated for Application in COVID-19 Patients

**DOI:** 10.3390/s20236774

**Published:** 2020-11-27

**Authors:** Francisco José Vivas Fernández, José Sánchez Segovia, Ismael Martel Bravo, Carlos García Ramos, Daniel Ruiz Castilla, José Gamero López, José Manuel Andújar Márquez

**Affiliations:** 1 Control and Robotics Research Team (TEP192), University of Huelva, 21004 Huelva, Spain; daniel.ruiz@diesia.uhu.es (D.R.C.); jose.gamero@diesia.uhu.es (J.G.L.); andujar@uhu.es (J.M.A.M.); 2 Structure of Matter Research Team (FQM318), University of Huelva, 21004 Huelva, Spain; jose.sanchez@dci.uhu.es (J.S.S.); imartel@uhu.es (I.M.B.); carlos.garcia@iesppg.net (C.G.R.)

**Keywords:** COVID-19, low-cost ventilator, lung infection, non-invasive ventilation, mechanical ventilation

## Abstract

Although the cure for the SARS-CoV-2 virus (COVID-19) will come in the form of pharmaceutical solutions and/or a vaccine, one of the only ways to face it at present is to guarantee the best quality of health for patients, so that they can overcome the disease on their own. Therefore, and considering that COVID-19 generally causes damage to the respiratory system (in the form of lung infection), it is essential to ensure the best pulmonary ventilation for the patient. However, depending on the severity of the disease and the health condition of the patient, the situation can become critical when the patient has respiratory distress or becomes unable to breathe on his/her own. In that case, the ventilator becomes the lifeline of the patient. This device must keep patients stable until, on their own or with the help of medications, they manage to overcome the lung infection. However, with thousands or hundreds of thousands of infected patients, no country has enough ventilators. If this situation has become critical in the Global North, it has turned disastrous in developing countries, where ventilators are even more scarce. This article shows the race against time of a multidisciplinary research team at the University of Huelva, UHU, southwest of Spain, to develop an inexpensive, multifunctional, and easy-to-manufacture ventilator, which has been named ResUHUrge. The device meets all medical requirements and is developed with open-source hardware and software.

## 1. Introduction

On March 11th, The World Health Organization (WHO) declared SARS-CoV-2 (COVID-19) a pandemic. As of 1 September 2020, the WHO had reported more than 4,500,000 confirmed cases in Europe and more than 220,000 deaths, almost 15% of which occurred in Spain [1,2]. 

While most patients are asymptomatic or suffer from mild symptoms (flu-like symptoms), approximately 10–15% of patients suffer from moderate or severe respiratory disorders, while approximately 5% of patients suffer very severe respiratory symptoms, which require admission to the critical care unit (CCU) [3,4]. These patients develop bilateral pneumonia or acute respiratory distress syndrome (ARDS), which causes an elevated pulmonary inflammatory process that leads to progressive deterioration/collapse of the lungs and to the death of the patient [5,6].

In the case of lung damage, the use of mechanical ventilation enables an increase in the amount of air and oxygen delivered to the patient through an increase in inspiratory pressure, which forces the oxygen to reach a higher proportion in the alveoli [7,8]. It has been demonstrated that the use of mechanical ventilation systems helps to increase the blood oxygen saturation to levels compatible with life, therefore a reduction in mortality [9,10].

The virulence of the pandemic has brought most healthcare systems to the brink of collapse, lacking healthcare personnel and essential medical equipment, especially mechanical ventilation [11,12]. This situation is even more dramatic in underdeveloped countries, which lack sufficient health structures and economic resources to face the pandemic.

With the aim of filling this gap, many research groups have contributed by offering design and implementation options for simple, low-cost ventilators that can be easily reproduced, which can guarantee a certain level of performance.

In the first instance, we can find models made with 3D designs and electrical motors that generate the airflow through a compression mechanism regulated in time and volume over an airway mask bag unit (AMBU) [13,14] or bag valve mask (BVM) [15,16]. These proposals are characterized by a simple, open-source, low-cost, and high reproducibility ventilator model. On the other hand, the lack of the regulation of necessary parameters to guarantee correct ventilation, as well as the lack of oxygenation in the supplied air make this type of equipment unusable in hospital care.

Secondarily, it is possible to find solutions based on the development of special 3D pipelines for the conversion of non-invasive equipment, usually used for the treatment of apnea or exacerbated chronic respiratory failure (EPOC), to invasive ventilators [17,18]. The main problem of this type of system lies in its reduced inspiratory flow capacity, the need for external air oxygenation, as well as the possibility of carbon build-up in the blood due to incorrect CO_2_ removal during expiration [19]. 

Finally, more complex models can be found, which are based on the use of gas pressure intake, implementing a pneumatic circuit of some complexity, which through the use of solenoid valves, enables the control of the direction of inspiratory and expiratory flow, as well as the applied pressure or inspired volume [20,21,22,23,24]. The main problem lies in the portability of these systems, increasing their size, the difficulty of transport, and the use if no compressed air intake is available on-site [25].

An example of the simplest configuration of this type of system can be found in [20], which presented a pneumatic system, making use of a reservoir bag, to increase the inertia of the system and reduce pressure changes. Other systems that are similar to the ones described above but with the possibility of oxygen injection were presented in [21,22,23]. In this sense, in [21], a reservoir bag was used to obtain oxygen-enriched air, which can be obtained directly from a pressurized intake with medical standards. In this case, there is no control or monitoring of the oxygen concentration, which will reach a default value. A more complex solution was presented in [23], in which a Venturi was used for the oxygen mixture to achieve a maximum concentration of 50%, which may be insufficient in the cases of the severe deterioration of the patient. Finally, the example presented in [22] enables the monitoring of the oxygen concentration in the air supplied to the patient, which requires an external combination of the equipment to guarantee the desired value.

Despite constituting high-performance solutions that technically allow the monitoring and control of certain respiratory parameters, none of them has been medically validated through animal testing.

Finally, it is important to emphasize that none of the solutions studied allow implementing more complex ventilation modes, such as the air pressure release ventilation (APRV) mode, which is showing significant improvements for the stabilization of COVID-19 patients [26,27].

Considering the shortcomings found in the scientific literature, as well as the urgent needs to address the deficiencies found in basic medical equipment, this article presents the design, development, testing, and validation of a low-cost, open-source, and high-performance pressure-controlled ventilator prototype, to be used in both non-invasive (NIV) and invasive ventilation. Attending to the cost of materials and assembly time, ResUHUrge requires a material cost of less than 500 € and an approximate manual assembly time of eight hours. An industrialization process would significantly reduce the material cost and the manufacturing time (this would mean tens of times less than commercial equipment with similar characteristics). Finally, it is important to note that the objective of this project was not to develop a commercial product that met the highest medical standards, but to provide a rapid technical solution that met the basic requirements for use in patients with moderate to severe respiratory failure. The paper is organized as follows: Section 2 explains the principle of operation of the developed ventilator. Section 3 describes the hardware architecture of the device, the proposed control algorithm, as well as the user interface, which will be further medically tested and validated in an artificial lung and in a test with an animal (following applicable Spanish regulations), in Section 4 and Appendix C, respectively. In both sections, the ventilator performance is discussed, highlighting the main aspects regarding its operation. Finally, in Section 5, the main conclusions of the research are addressed. 

## 2. Operating Principle

ResUHUrge is a BiPAP (bi-level positive airway pressure)-type ventilator, with independent regulation of intrathoracic pressure, i.e., one level during inspiration, IPAP (inspired positive airway pressure), and another during expiration, EPAP (expired positive airway pressure) [8,28]. The difference between IPAP and EPAP is known as pressure support ventilation (PSV). The possibility of working with different pressures between EPAP and IPAP is fundamental, since it allows the clinician to increase ventilation per minute, thus achieving adequate tidal volumes demanded by the patient and avoiding pulmonary barotrauma [8,29]. The ventilator works by generating a constant flow according to the maximum pressure selected (up to 35 cm H_2_O). This flow is regulated by a solenoid valve that diverts the flow towards the patient, regulating both the maximum inspiratory pressure (IPAP) and the pressure at the end of expiration (EPAP).

For the correct regulation of operating pressures, IPAP and EPAP, depending on the needs and breathing times of the patient, it is necessary to define the conditions that determine the transition between inspiration and expiration cycles. These conditions are defined as inspiratory trigger and cycling [30,31].

The inspiratory trigger is defined as the condition that allows determining the beginning of inspiration and the end of expiration, and therefore defines the expiration time ($Exp_{Time}$) and the imposition of the IPAP pressure setpoint [16,30]. On the other hand, the cycling condition determines the end of inspiration and the beginning of expiration, that is, it defines the inspiration time (*Ins*$p_{Time}$) and the imposition of the EPAP pressure setpoint [16,30]. Both conditions can be given by airflow (assisted mode) or by time (controlled mode).

**Figure 1:**
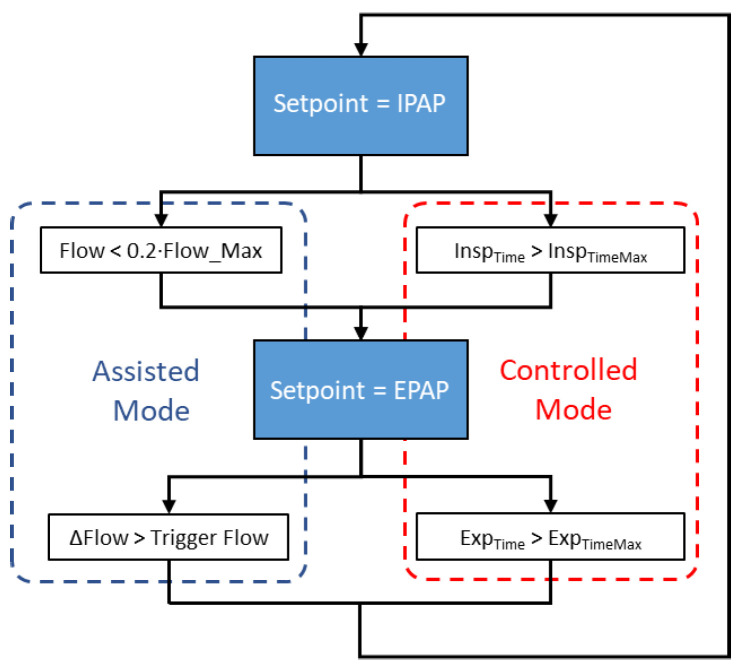
Flow diagram of the ventilator operating mode.

Based on the inspiratory trigger and cycling conditions, ResUHUrge allows two modes of ventilation to be implemented as needed, assisted mode and controlled mode; see Figure 1.

Assisted mode is characterized because in this case, the patient determines the respiratory cycle and the ventilator assists the patient throughout the breathing cycle, providing the required airflow. The ventilator provides bi-level pressure support, activating positive pressure (IPAP) in response to spontaneous inspiratory effort and cycles to positive expiratory pressure (EPAP) during expiration.

**Figure 2:**
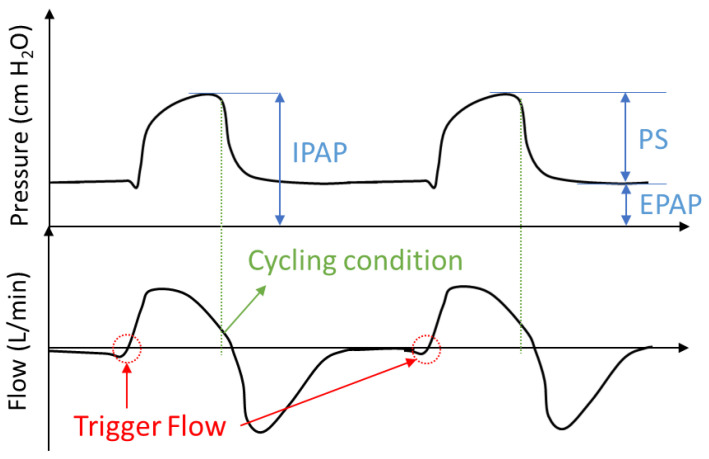
Example of the trigger and cycling condition in assisted mode.

At the onset of inspiration, the patient makes an inspiratory effort that slightly reduces intrathoracic pressure and increases inspiratory flow with a high positive value slope [30]. Accordingly, the inspiratory trigger condition is defined by a value of the derivative of inspiratory flow equal to or greater than the sensitivity setting (trigger level), activating the inspiration phase (Figure 2) [31,32].

The trigger level can be selected according to the patient’s respiratory conditions so that the selected value maintains a compromise between vulnerability to false triggers and the inspiratory effort required by the patient, which may result in the patient failing to synchronize with the ventilator [16,33].

The cycling variable ends the inspiratory phase. A breath can be cycled by one of several variables including time, flow, volume, or pressure [30,34]. In this case, the cycling condition is defined as a percentage of the peak flow level reached during the inspiratory process (usually between 20 and 30%); see Figure 2. This condition determines the end of the inspiratory process, establishing enough time frame for the pressure setpoint change due to impending lung saturation.

Finally, for safety reasons, if the patient does not initiate a spontaneous breath in the time determined by the alarm control (apnea), 10 s, the ventilator starts a time-triggered and cycled breath (inspiratory time), limited by the pre-set IPAP level, initiating controlled mode.

On the other hand, controlled mode (Figure 1) is used when the patient is not able to support breathing by himself/herself. Unlike assisted mode, this mode is characterized by time-controlled cycles and triggers that guarantee patients a minimum or desired number of breaths based on the programmed or maximum inspiratory/expiratory times (Equations (A1) and (A2), respectively, Appendix A), selected according to the patient’s requirements for inspired volume and oxygen saturation [16,35]. 

Time-cycling can be used, for example, when the patient is sedated or cannot breathe on his/her own. In fact, once the clinician has selected the trigger level, the ventilator automatically carries out the breathing control, thus ensuring the patient’s ventilation.

Finally, within controlled operation mode, the device allows the implementation of APRV ventilation mode, by choosing the required inspiration-expiration ratio.

## 3. Developed Ventilator

### 3.1. System Architecture

**Figure 3:**
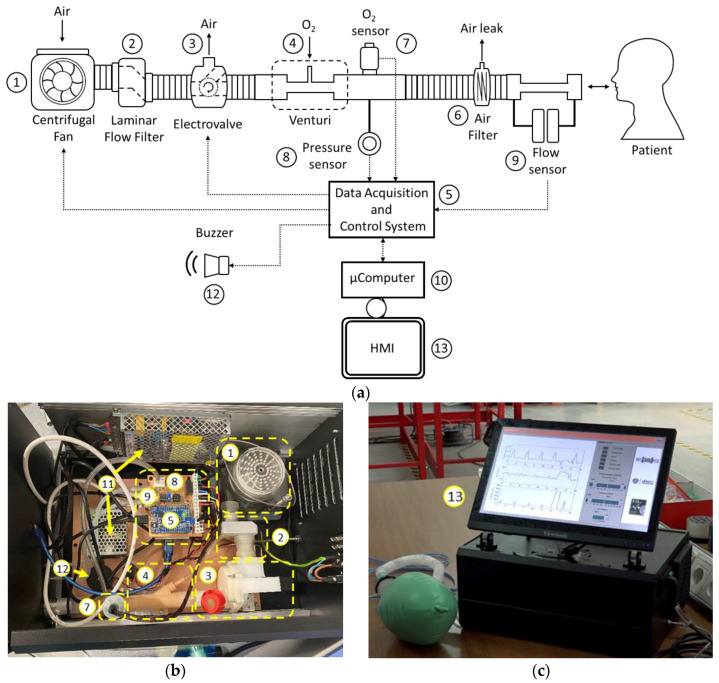
Architecture of ResUHUrge: (**a**) general scheme; (**b**) ResUHUrge interior detail and (**c**) touch screen detail.

The main element of the ventilator is a high-flow, high-static pressure centrifugal fan (1, Figure 3), with enough capacity to meet the needs in terms of the mass flow for patients with respiratory distress. The regulation of the fan is based on a three-phase speed PWM controller for brushless motors, which is programmed to produce the maximum airflow, maintaining a constant regime at all times, which is crucial to increasing the lifetime of the fan. To reduce the disturbances associated with a turbulent flow from the fan, a laminar flow filter is used (2, Figure 3).

**Figure 4:**
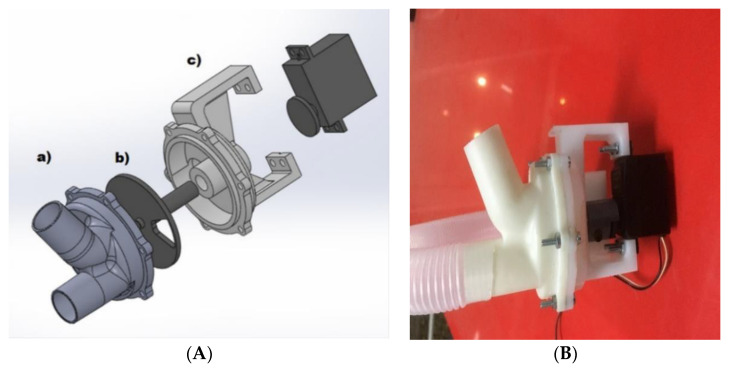
Developed three-way solenoid valve (**A**) 3D design: (a) main body detail; (b) rotating stem detail and (c) detail of the servo support and air chamber; (**B**) final appearance.

Airflow control is accomplished by using a low-cost three-way resin solenoid valve designed specifically for the application (3, Figure 3). The design of this solenoid valve is based on the use of a controlled rotating element and a watertight chamber with an inlet (centrifugal fan) and two outputs (patient and outside) specially designed to reduce the disturbances in the mass flow during the transitions from one state to another (Figure 4). 

This design allows the total or partial airflow generated by the turbine to be diverted either to the patient or to the outside depending on the opening of the valve. As a valve control element, a low-cost, high-speed servomotor PWM with high static torque is used. The use of servomotors with high static torque allows reducing the disturbances in the output flow caused by air injection from the fan. The relative position of the servomotor will allow defining the percentage of mass flow, thus allowing the control of airflow and pressure delivered to the patient, according to the control loop, which will be explained later.

**Figure 5:**
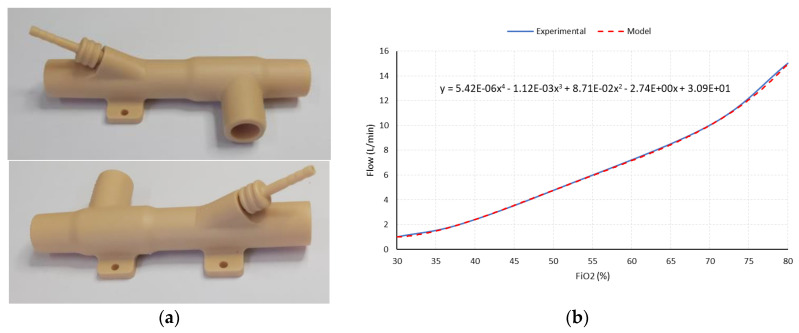
Venturi: (**a**) image; (**b**) FiO_2_ as a function of flow.

In order to satisfy the patient’s oxygen saturation needs, the use of active oxygenation by means of medical oxygen may be required. To this end, a Venturi (4, Figure 3) is used, which produces negative pressure at its inlet, which allows passive suction and homogeneous mixing of oxygen from a typical low-pressure oxygen inlet, resulting in a mixture of oxygen-enriched air. The Venturi used is based on a resin design (Figure 5a), specially designed according to the characteristics of the flow generated, which allows a maximum FiO_2_ of 80% to be guaranteed (Figure 5b).

The channeling of enriched air to the patient is done by means of a medical-grade corrugated PVC tube, which incorporates a bacterial-viral filter to avoid contamination of the device and channeling (6, Figure 3). Finally, the filter incorporates an air leak line, which is fundamental and necessary to guarantee the air evacuation and CO_2_ elimination during the patient’s exhalation, due to the use of positive EPAP pressures.

For the correct management and control of the ventilator, an acquisition and control system was designed (5, Figure 3). This system is able to condition and read the analog output variables of the different sensors, as well as the implementation of the control logic, communications, and alarm management required.

In order to ensure proper operating conditions at all times, and implement the control loops, the ventilator incorporates sensors to measure oxygen concentration (7, Figure 3), as well as the mass flow (9, Figure 3) and air pressure of the patient’s supply circuit (8, Figure 3). The output of the sensors used is represented by a differential low voltage amplitude analog signal. The main characteristics of the sensors used are shown in Table 1.

**Figure 6:**
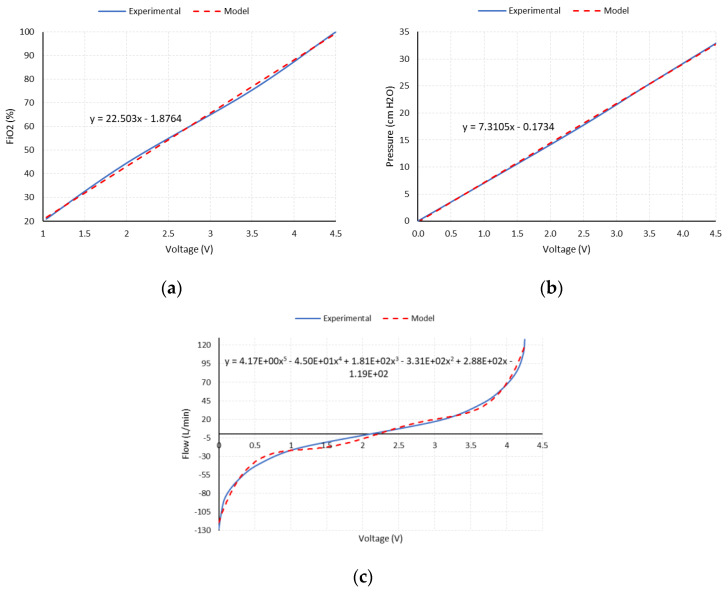
The amplified analog output of the sensors and model used. (**a**) Oxygen sensor; (**b**) pressure sensor; (**c**) airflow sensor.

The conditioning circuit of the sensors’ output signals is based on high-performance, high-gain instrumentation amplifiers, Model INA126P from the manufacturer Texas Instruments^®^. The gain of each instrumentation channel is selected according to the maximum expected output of its sensor and the input range of the analog-to-digital converter of the microcontroller used (0–5 VDC). Figure 6a,b show the amplified analog output of the oxygen concentration and pressure sensors, along with the linear expression used for their modelling, respectively.

For the specific case of the flow sensor, a Hamilton Medical^®^ flow sensor and differential pressure sensor are used. The amplified output is reconditioned to allow the measurement of inspiratory (positive) and expiratory (negative) flow values. For this purpose, a zero and gain correction circuit is used, using operational amplifiers, Model OPA2227 from the manufacturer Texas Instruments^®^, in a differential configuration, so that the output signal of the amplifier is superimposed on a continuous 2.5 VDC component, meaning that the limit values of the sensor (positive and negative maximums) coincide with the limits defined by the measurement range of the microcontroller’s ADC (0–5 VDC). Figure 6c shows the amplified analog output of the flow sensor along with the polynomial expression used for its modelling.

The ATmega2560 microcontroller was used. The programming of this microcontroller was entirely developed in the Arduino IDE environment.

The control of ResUHUrge was performed by using an HMI consisting of a specifically developed graphical user interface (GUI) and buzzers for audible alarms (12, Figure 3), which can be managed through its integrated 22″ touch screen (13, Figure 3) or through its integration with a local network or an Internet connection (through an Ethernet cable or WiFi), even being able to become its own WiFi network and independent Internet hotspot. Its WiFi communication capabilities enable a full remote control of the ventilator, as well as the visualization of all data, so the presence of medical staff with the patient to manage and consult the ventilator is not required, which will reduce infection possibilities and decrease the time necessary to deliver care.

The touch screen control, the communication management (via Ethernet cable or WiFi), as well as the interface between the GUI and the acquisition and control system are based on the use of a Raspberry Pi microcomputer (10, Figure 3). 

**Figure 7:**
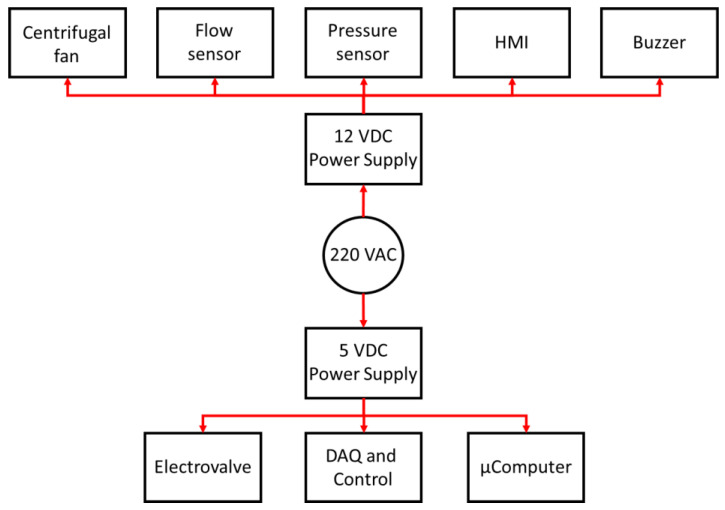
Power supply architecture of ResUHUrge.

Finally, according to the power requirements of the different devices that make up ResUHUrge, two low-cost medical-standard 5 VDC and 12 VDC power supplies are used (11, Figure 3). Figure 7 shows the general power supply architecture of the ResUHUrge prototype.

The main characteristics of the devices that make up the ventilator are listed in Table 1.

### 3.2. Pressure Controller

ResUHUrge is a BPAP ventilator, and therefore, its operation is based on pressure regulation, enabling the assistance or control of the patient’s breathing cycle. This is done by setting the pressure setpoint according to the respiratory phase, IPAP for inspiration and EPAP for expiration.

This regulation is established through the control of a three-way solenoid valve, whose stem position is determined by the servomotor. In this way, the angle of rotation of the servomotor will determine the opening of the channel and therefore the amount of airflow delivered to the patient and the outside, and indirectly the pressure of the patient line (see 3 in Figure 3). For the regulation of pressure and control of the solenoid valve, a PID controller is used. Appendix B includes the PID controller design and the system pressure control scheme. 

### 3.3. User Interface

In order to monitor the patient’s respiratory parameters, while allowing the regulation of working pressure, operating modes, etc., ResUHUrge has a very intuitive GUI, programmed in Python, and specifically designed for the application to comply with the requirements of medical specialists in mechanical ventilation. 

The GUI (13, Figure 3) consists of two panels, the control panel, where the main respiratory variables are controlled and monitored, and the alarm panel, from which the critical values of the parameters are detected and configured according to the patient and ventilation requirements.

**Figure 8:**
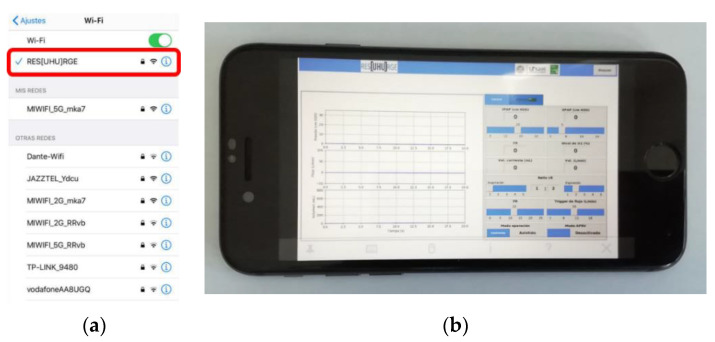
Remote controller of the developed ventilator. (**a**) WiFi connection point; (**b**) mobile app.

Figure 8 shows the remote connection point and the app designed for mobile phones.

#### 3.3.1. Control Panel

**Figure 9:**
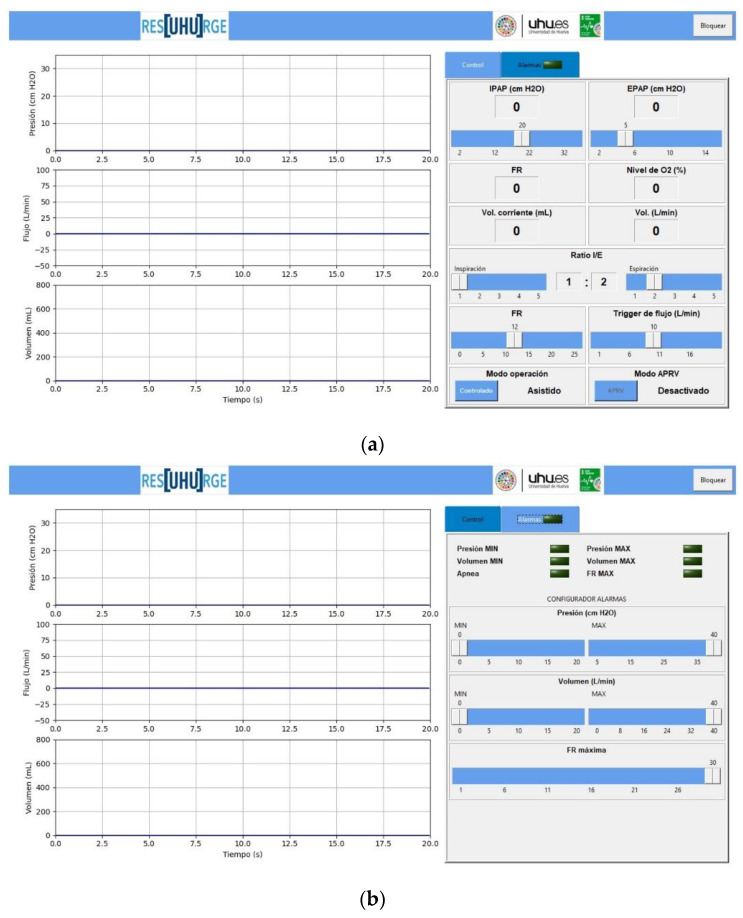
GUI of the developed ventilator. (**a**) Control panel; (**b**) alarm panel.

The control panel represents the main panel of the GUI (Figure 9a) from which the specialist can continuously monitor the patient’s respiratory pressure and flow (current value and parameter waveform) and interact in a very ergonomic way with the ventilator, controlling and selecting parameters such as IPAP pressure (cm H_2_O), EPAP pressure (cm H_2_O), I/E ratio, according to Equation (A3), Appendix A, inspiration trigger (L/min), and time-based cycling and trigger, Equations (A1) and (A2), Appendix A.

Similarly, controls are available for the selection of ventilation modes (assisted or controlled). Within the functionality of the controlled ventilation mode, the APRV ventilation function is allowed (see Figure 9a).

In addition to the previous controls, the GUI also shows other very useful data, such as the actual ventilation mode, the real IPAP and EPAP value, the patient’s breathing frequency (BF), according to Equation (A4), Appendix A, the I/E ratio, the tidal volume or volume inspired by each inspiration (mL), according to Equation (A5), Appendix A, the volume of inspired air (L/min), Equation (A6), Appendix A, the percentage of oxygen supplied to the patient, from 21% in a normal atmosphere to that prescribed by the clinician, the real-time graph of the respiratory pressure (cm H_2_O), the respiratory flow (L/min), and the respiratory volume (L) (see Figure 9a). Appendix A contains the formulation for the calculation of ventilatory parameters.

#### 3.3.2. Alarm Panel

The alarm panel is an important function in terms of safety and comfort for both patients and medical staff. In this sense, ResUHUrge has been designed to guarantee safe conditions for the patient and therefore integrates analysis logic to detect and configure alarm conditions related to the main respiratory variables: the maximum and minimum breathing pressure (which can be configured), the minimum and maximum inspiratory volume, tidal volume, and inspired volume per minute (can be configured), the maximum BF (can be configured), or if, on the contrary, the patient presents apnea, that is, it detects if there is no breathing in a time interval defined by the clinician (see Figure 9b).

Finally, Table 2 summarizes the main functional characteristics of the ResUHUrge ventilator.

## 4. Ventilator Performance

In order to validate the performance of the ResUHUrge prototype, several experimental tests were conducted. All of them were carried out following the applicable Spanish regulations.

**Figure A2:**
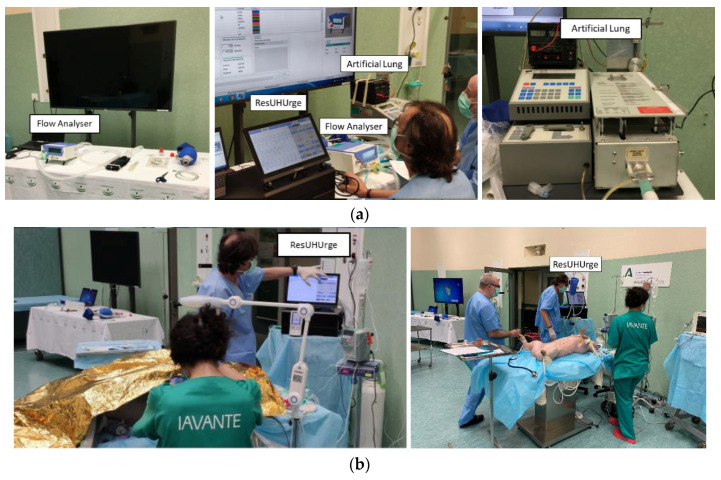
(**a**) Calibration test and artificial lung test; (**b**) animal test.

Firstly, the developed acquisition system was validated and calibrated by comparing the values of the main respiratory variables with a professional flow analyzer Model Flow LAB PF-302 from the manufacturer IMT Medical^®^, used for calibrating high-performance commercial ventilators (Figure A2, Appendix C). Subsequently, the behavior of the ventilatory modes was tested and validated on a ventilator tester (professional artificial lung) Model VT-2 from the manufacturer BIOTEK^®^, which allows the simulation of any degree of respiratory compliance and resistance related to the degradation of a patient’s respiratory capacity (Figure A2, Appendix C). 

In this case, a total of eight tests were performed with different ventilatory conditions, compliance values, and airway resistance, with the aim of validating the operation of the developed ventilator against different patient conditions. For each test, ResUHUrge was programmed with different ventilatory parameters and compared with the average values obtained from the professional flow analyzer (Flow LAB) for tests lasting three minutes. Table 3 shows the results obtained for the different experimental tests.

In this case, the professional flow analyzer was used to simulate different levels of static compliance, associated with lung elasticity. In the different experimental tests, simulations were made to validate the behavior of ResUHUrge in the case of the ventilation of healthy lungs (50 mL/mbar), as well as lungs affected by severe ARDS (≤30 mL/mbar).

On the other hand, several airway resistance values were used, with the aim of simulating different patient conditions, from low resistance (5 mbar/L/s) to high values (≥20 mbar/L/s), corresponding to high obstruction.

Finally, in Tests 7 and 8, validation tests of APRV mode under adverse ventilatory conditions were performed.

On the other hand, and following the applicable Spanish regulations, the medical validation of the device was performed through animal testing. Appendix C describes the development of the animal test carried out and includes the results obtained in arterial blood gasometry.

### Discussion

According to the ventilatory conditions (compliance and airway resistance), we can distinguish between two types of tests (see Table 3), healthy lung tests (1 and 4) and lung tests in case of distress (2, 3, and 5 to 8), characterized by high compliance and reduced airway resistance and low compliance and high airway resistance, respectively.

In the first instance, for the most favorable cases (Tests 1 and 4) and cases with moderate to severe distress and medium PSV (Tests 2, 7, and 8), ResUHUrge demonstrated sufficient capacity to ventilate the simulated patient, reaching the required IPAP and EPAP pressures, with high values of inspired volume (~4–5 L, Tests 1 and 4). In particular, Tests 3 and 7–8 showed the effectiveness of ResUHUrge in the ventilation of a very critical patient (compliance 10 mL/mbar and resistance >50 mbar/L/s), and the correct application of APRV mode (identified by the 12/1 I/E ratio), even in cases of high airway resistance. The values of the imposed ventilatory parameters were validated by the measurements of the Flow LAB analyzer (Table 3).

Finally, it is important to emphasize simulation in the worst ventilation conditions (Tests 5 and 6). In these cases, the high PSV required and the demanding conditions of the patient (reduced compliance and high resistance) mean that the centrifugal fan does not have sufficient ventilatory capacity to reach the maximum pressure (which, if necessary, would be very easy to solve simply by changing the centrifugal fan for a higher power one), and therefore, close maximum values are reached, depending on the ventilatory conditions of the simulated patient (IPAP = 28.5 cm H_2_O and IPAP = 25.5 cm H_2_O for Tests 5 and 6, respectively). According to medical criteria and recommendations, the maximum IPAP conditions reached in these critical cases can be considered sufficient for the ventilation of critical patients, since the use of very high pressures can be harmful because of the risk of creating barotraumas.

In view of the above, it was proven that ResUHUrge is capable of ventilating patients even in cases of extreme pulmonary deterioration, characterized by very low compliance values and very high airway resistance, responding even in the most unfavorable cases. These results were validated by comparison with the values obtained by a professional flow analyzer (Flow LAB), with a measurement error within the tolerance range for this type of device.

On the other hand, according to the test carried out with an animal (see Appendix C), the use of ResUHUrge increased the level of oxygenation in the blood, while reducing the concentration of CO_2_ in the blood and expired air to standardized values. This is clearly reflected in the results of the gasometry. 

Regardless of the animal’s condition, ResUHUrge demonstrated the ability to maintain proper ventilation at all times, easy operation, accessibility to adjustment controls, and easily understandable alarm system limits.

## 5. Conclusions

At the height of what is known as the second wave of COVID-19, pharmaceutical laboratories are fighting against time in the development of a vaccine against the virus. Until that happens, it is important to have personnel resources and medical material to deal with the virus until the vaccine becomes a reality. 

In order to cope with the most severe symptoms caused by the SARS-CoV-2 virus, the use of mechanical ventilators has proven to be an effective tool in the treatment of ARDS, increasing the probability of survival in the most critical cases.

In response to the lack of medical equipment, this article presents a ventilator device prototype, ResUHUrge, which has been developed to meet all necessary medical criteria for use even in intensive care units. ResUHUrge is a BIPAP, pressure-controlled-type mechanical ventilator that allows the monitoring and control of the main respiratory parameters, including airway pressure (up to a maximum pressure of 28 cm H_2_O in this unit, but that can be easily increased by changing the centrifugal fan in later versions), airflow to the patient, inspiratory volume, breathing frequency, FiO_2_ (up to 80% in this unit, but that can be easily increased by redesigning the Venturi in later versions), and the I/E ratio, among others. 

Its high flow capacity, easy handling, ergonomics, remote connectivity, and the implementation of additional functions, such as APRV mode, make ResUHUrge stand out as a fully functional and highly technological prototype compared to prototypes proposed in the scientific literature. 

The experimental results obtained from the tests with a professional artificial lung and flow analyzer, and finally with an animal, showed the excellent performance of the device. Additionally, it was medically validated and supported by the intensive care unit of the Juan Ramón Jimenez University Hospital, Huelva (southwest of Spain).

In particular, the excellent characteristics of ResUHUrge and its low cost would enable the production of a sufficient number of ventilators to deal with respiratory diseases (such as COVID-19, among others) in their initial stages, before entering the ICU, and before endotracheal intubation. The non-invasive ventilation (NIV) enabled by ResUHUrge is applicable to both acute and chronic patients. NIV offers important advantages, such as the possibility of avoiding intubation and invasive ventilation with their potential complications. It is also more comfortable for the patient, does not require deep sedation, and allows the defense mechanisms of the upper airway to be preserved. In patients with acute respiratory failure, NIV reduces tracheal intubation, morbidity, mortality, and hospital stay. In chronic patients, it can improve symptoms, quality of life, and certain physiological parameters.

In summary, ResUHUrge is presented as a low-cost, high-performance ventilator that can be used in COVID-19 patients and others. It is especially indicated for use in respiratory diseases, where one of the best treatments for patients is the best possible ventilation.

## Figures and Tables

**Table 1:** Main parameters of the ventilator components.

Element	Model	Characteristics
Centrifugal fan	WM7040, Steady Motor^®^	Input voltage: 12 V
		Idle speed: 36,000 rpm
		Idle current: 2.8 A
		Maximum air volume: 140 L/min
		Maximum wind pressure: 7.5 kPa
		Blower type: brushless DC
Electronic speed control	Aerostar 20A, Aerostar^®^	Input voltage: 6–15 VDC
		Output current: 20 A (continuous)
Servo	FUTM0031, Futaba^®^	Input voltage: 5 VDC
		Torque: 3.17 kg∙cm
		Speed: 0.23 s/60°
		Modulation: Analog
Microcontroller	ATmega2560, Microchip^®^	Input voltage: 5 VDC
		Architecture: 8-bit AVR RISC
		CPU speed: 16 MHz
		Program memory size: 256 kB
		SRAM: 8 kB
		EEPROM: 4 kB
		ADC: 16 inputs (10 bits)
O_2_ sensor	OOM111, Honeywell^®^	Measurement range: 0–100%
		Output voltage: 0.6 mV/%
Pressure sensor	SM1297-008, Silicon Microstructures^®^	Flow range: 55 cm H_2_O
		Excitation voltage: 8–15 VDC
		Output voltage: 1.1 mV/cm H_2_O
Flow sensor	AWM92100, Honeywell^®^	Flow range: ±200 SCCM
		Excitation voltage: 8–15 VDC
		Output voltage: ±80 mV (non-linear)
Microcomputer	Raspberry Pi 4 Model B	Processor: Broadcom BCM2711
		SDRAM: 8 GB LPDDR4-3200
Buzzer	5117636, RS Pro^®^	Input voltage: 3–16 VDC
		Sound output: 90 dB
5 VDC power supply	SGS-25-5, RS Pro^®^	Output voltage: 5 VDC
		Rated power: 25 W
12 VDC power supply	SRS-150-12, RS Pro^®^	Output voltage: 12 VDC
		Rated power: 150 W
Touchscreen monitor	TD2220, ViewSonic^®^	Resolution: 1920 × 1080
		Standing screen display size: 22″

**Table 2:** ResUHUrge specifications.

Element	Characteristics	
Respiratory capacities	Pressure: 30 cm H_2_O	I/E ratio: 20~100%
	Flow per minute: 0~140 L/min	FiO_2_: 20~80%
Controlled parameters	Operating mode (assisted/controlled)	Minimum pressure alarm level
	Flow trigger	Maximum pressure alarm level
	I/E ratio	Minimum inspired volume
	Breathing Frequency	Maximum inspired volume
	APRV	Maximum breathing frequency
Monitored parameters	IPAP (cm H_2_O)	Inspired volume (L/min)
	EPAP (cm H_2_O)	I/E ratio
	FiO_2_ (%)	Breathing frequency
	Tidal volume (L)	
Alarms	Minimum pressure	Maximum inspired volume
	Maximum pressure	Maximum breathing frequency
	Minimum inspired volume	Apnea
Interface	Touch screen	
	Ethernet cable WiFi	

**Table 3:** ResUHUrge and Flow LAB ventilation parameters for different simulation test.

				Programmed (ResUHUrge)/Measured (Flow LAB)					
Test	Ventilator Unit	Lung Parameters		IPAP (cm H_2_O)	EPAP (cm H_2_O)	BF	I/E	$Vol_{insp}$ (mL)	$Vol_{min}$ (L/min)
1	ResUHUrge	Compliance: 50 mL/mbar Resistance: 5 mbar/L/s	↑	15	5	12	1/2	350	4.20
	Flow LAB		↓	14.8	5.9	11.9	1/2	370	4.40
2	ResUHUrge	Compliance: 20 mL/mbar Resistance: 20 mbar/L/s	↓	15	5	12	1/2	255	3.06
	Flow LAB		↑	14.7	5.7	11.9	1/2	261	3.10
3	ResUHUrge	Compliance: 10 mL/mbar Resistance: 50 mbar/L/s	↓	15	5	12	1/2	23	0.28
	Flow LAB		↑	14.8	5.7	12.8	1/1.8	27	0.34
4	ResUHUrge	Compliance: 50 mL/mbar Resistance: 5 mbar/L/s	↑	30	5	12	1/2	426	5.11
	Flow LAB		↓	29.8	5.5	12.1	1/1.8	445	5.38
5	ResUHUrge	Compliance: 20 mL/mbar Resistance: 20 mbar/L/s	↓	30	5	12	1/2	258	3.10
	Flow LAB		↑	28.5	5.5	12.4	1/1.9	253	3.13
6	ResUHUrge	Compliance: 10 mL/mbar Resistance: 50 mbar/L/s	↓	30	5	12	1/2	219	2.63
	Flow LAB		↑	25.5	5.5	12.4	1/1.9	225	2.79
7	ResUHUrge	Compliance: 30 mL/mbar Resistance: 20 mbar/L/s	↑	15	5	14	12/1	79	1.11
	Flow LAB		↑	15.8	4.3	14.9	11.8/1	66	0.98
8	ResUHUrge	Compliance: 30 mL/mbar Resistance: 20 mbar/L/s	↑	25	10	14	12/1	88	1.23
	Flow LAB		↑	24.7	8.6	14.9	11.8/1	93	1.39

**Figure A1:**
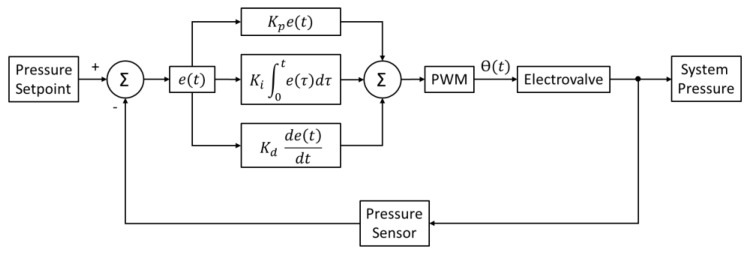
System pressure PID controller.

**Figure A3:**
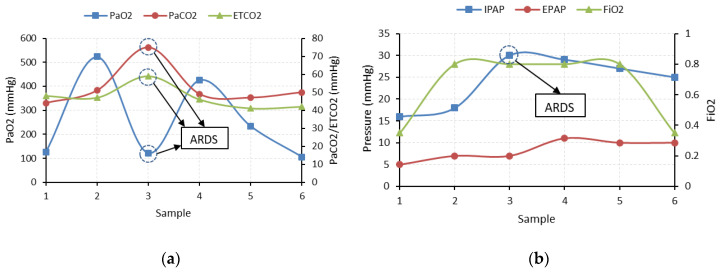
Animal test: (**a**) PaO_2_, PaCO_2_, and ETCO_2_; (**b**) IPAP, EPAP, and FiO_2_.

## List of Acronyms

ADC	analog to digital converter
AMBU	airway mask bag unit
APRV	airway pressure release ventilation
ARDS	acute respiratory distress syndrome
BiPAP	bilevel positive airway pressure
BVM	bag valve mask
CPAP	continuous positive airway pressure
DAQ	data acquisition system
EPAP	expiratory positive airway pressure
HMI	human-machine interface
ICU	intensive care unit
IPAP	inspiratory positive airway pressure
I/E	inspiration-expiration ratio
GUI	graphical user interface
NIV	noninvasive ventilation
PEEP	positive end-expiratory pressure
PSV	pressure support ventilation
PWM	pulse width modulation
WHO	World Health Organization

## Notation and Symbols

BF	breathing frequency (breaths per minute)
$BF_{Setpoint}$	desired breathing frequency (breaths per minute)
$E_{Rate}$	expiration rate from I/E ratio: APVR mode (1)
$Exp_{Time}$	expiration time (s)
$Exp_{TimeMax}$	maximum expiration time (s)
$Insp_{Time}$	inspiration time (s)
$Insp_{TimeMax}$	maximum inspiration time (s)
$I_{Rate}$	inspiration rate from I/E ratio, APVR mode (12)
I/E ratio	ratio between inspiration and expiration time
$T_{s}$	sample period (s)
$VoL_{insp}$	tidal volume or inspired volume in each inspiration (mL)
$VoL_{min}$	inspired volume per minute (L/min)

## Appendix A. Ventilatory Parameters

### Appendix A.1. Maximum Inspiration Time

(A1) $$Insp_{TimeMax}=\frac{60}{BF_{Setpoint}}\cdot \frac{I_{Rate}}{I_{Rate}+E_{Rate}}\;$$

where:

$Insp_{TimeMax}$: maximum inspiration time (s).

$BF_{Setpoint}$: desired breathing frequency (breaths per minute).

$I_{Rate}$: inspiration rate from I/E ratio. In APRV mode, its value is 12.

$E_{Rate}$: expiration rate from I/E ratio. In APRV mode, its value is one.

### Appendix A.2. Maximum Expiration Time

(A2) $$Exp_{TimeMax}=\frac{60}{BF_{Setpoint}}\cdot \frac{E_{Rate}}{I_{Rate}+E_{Rate}}$$

where:

$Exp_{TimeMax}$: maximum expiration time (s).

### Appendix A.3. I/E Ratio

(A3) $$I/E\;ratio=\frac{Insp_{Time}}{Exp_{Time\;}}$$

where:

I/E ratio: ratio between inspiration and expiration time.

### Appendix A.4. Breathing Frequency

(A4) $$BF=\frac{60}{\left(Insp_{Time}+Exp_{Time}\right)}$$

where:

$Insp_{Time}$: inspiration time (s).

$Exp_{Time}$: expiration time (s).

BF: breathing frequency (breaths per minute).

### Appendix A.5. Tidal Volume

(A5) $$Vol_{insp}=\int_{0}^{Insp_{Time}}Flow\left(t\right)\;dt$$

where:

$Vol_{insp}$: tidal volume or inspired volume in each inspiration (mL).

### Appendix A.6. Volume per Minute

(A6) $$Vol_{min}=BF\cdot Vol_{insp}$$

where:

$Vol_{min}$: inspired volume per minute (L/min).

## Appendix B. PID Calculation

Equation (A7) shows the general formulation of a continuous-time PID controller.

(A7) $$u\left(t\right)=K_{p}\left(e\left(t\right)+\frac{1}{T_{i}}\int_{0}^{t}e\left(t\right)\;dt+T_{d}\cdot \frac{de\left(t\right)}{dt}\right)$$

where, in this case:

u(t) = control signal for solenoid valve.

e(t) = error in the patient’s line pressure.

$K_{p},T_{i},\;T_{d}$: PID parameters.

The designed control loop makes use of the error in the patient’s line pressure (e(t)), calculated from the pressure setpoint (IPAP or EPAP, depending on the respiratory cycle), and the pressure value measured through the installed sensor.

Finally, attending to the servomotor used, the controller output is represented by the duty cycle of a 50 Hz PWM signal, which determines its rotation angle. Figure A1 shows the system pressure controller scheme.

In order to be able to apply the proposed PID controller, it is necessary to discretize Equation (A7) so that it can be programmed in the microcontroller. Based on the above, discretizing the previous expression using the forward Euler method, (A7) is written for the *k*-th sample as (A8).

(A8) $$u\left(k\right)=K_{p}\left(e\left(k\right)+\frac{T_{s}}{T_{i}}\overset{k-1}{\underset{j=0}{\sum }}e\left(j\right)+\frac{T_{d}}{T_{s}}\left(e\left(k\right)-e\left(k-1\right)\right)\right)$$

where:

$T_{s}$: sampling period (s).

$K_{p},T_{i}$ and $T_{d}$: PID parameters to adjust.

From Expression (4) and its evaluation in the previous sampling period, a recursive expression is obtained that allows the calculation of the discrete PID, Equation (A9).

(A9) $$u(k)=u\left(k-1\right)+K_{p}\left(e\left(k\right)-e\left(k-1\right)+\frac{T_{s}}{T_{i}}e\left(k-1\right)+\frac{T_{d}}{T_{s}}\left(e\left(k\right)-2e\left(k-1\right)+e\left(k-2\right)\right)\right)$$

The parameters were adjusted experimentally, following the criteria of experienced doctors, according to the desired response time under different pressure setpoints and patient conditions. The parameter values of the designed discrete PID are listed in Table A1.

**Table A1:** PID parameters.

Parameter	Characteristics
$T_{s}$	0.01 s
$K_{p}$	35
$T_{i}$	0.06
$T_{d}$	0.03

## Appendix C. Animal Test

This test consisted of using the developed ventilator as a ventilatory support system applied to a pig under a normal lung condition (assisted mode) and severe distress induced (controlled mode) by means of a pulmonary washing with 2800 mL of physiological serum 0.9%.

In order to check the effects of mechanical ventilation on the gas concentration in blood, pleural probes were used to perform arterial gasometry every 30 min. The parameters under study were related to the concentration and partial pressures of O_2_ and CO_2_ in the blood, which determine the correct oxygenation during inspiration and the correct elimination of carbon dioxide during expiration. In this case, to measure the level of oxygenation in the blood, the arterial pressure of O_2_ in the blood (PaO_2_) is used as the reference parameter. For the concentration of CO_2_, the blood pressure of CO_2_ (PaCO_2_) and the concentration of CO_2_ in exhaled air (ETCO_2_) are studied. The results of the gasometry and the ventilatory parameters used in the animal test are shown in Figure A3a,b, respectively.

Once the distress was induced (Sample 3, Figure A3), the arterial oxygen pressure was drastically reduced (PaO_2_ from 520 mmHg to 110 mmHg), while there was a considerable increase in the concentration of CO_2_ in the blood and expired air (PaCO_2_ from 42 mmHg to 75 mmHg and ETCO_2_ from 47 mmHg to 59 mmHg), which reflects a reduction in the capacity of alveolar gas exchange and therefore lung degradation. To counteract this effect, ventilation with high FiO_2_ levels and IPAP pressures (0.8, 30 mbar respectively) was performed in order to compensate for the loss of lung capacity (Samples 4 and 5, Figure A3b). In successive samples, the recovery of the blood oxygenation pressure level (PaO_2_ > 100 mmHg) and the reduction of the CO_2_ concentration (PaCO_2_ ≤ 50 mmHg, ETCO_2_ ≤ 45 mmHg) can be checked, indicating the recovery of the correct gas levels in the animal (Samples 4 and 5, Figure A3).

Finally, prior to the euthanasia of the animal, the FiO_2_ was reduced, with the aim of analyzing the recovery of the pig’s respiratory capacities. According to the results (Sample 6, Figure A3, irreversible damage to the lungs was reflected by a reduction of blood oxygen pressure values to pre-distress levels, but with the use of high IPAP support pressure (PaO_2_ = 105 mmHg) and a slight increase in pressures and CO_2_ concentrations in blood and expired air (PaCO_2_ = 50 mmHg and ETCO_2_ = 42 mmHg).
